# Superabsorbent Hydrogels Based to Polyacrylamide/Cashew Tree Gum for the Controlled Release of Water and Plant Nutrients

**DOI:** 10.3390/molecules26092680

**Published:** 2021-05-03

**Authors:** Heldeney Rodrigues Sousa, Idglan Sá Lima, Lucas Matheus Lima Neris, Albert Santos Silva, Ariane Maria Silva Santos Nascimento, Francisca Pereira Araújo, Rafael Felippe Ratke, Durcilene Alves Silva, Josy Anteveli Osajima, Leilson Rocha Bezerra, Edson Cavalcanti Silva-Filho

**Affiliations:** 1 LIMAV, Interdisciplinary Laboratory for Advanced Materials, Piaui Federal University, Campus Universitário Ministro Petrônio Portella, Teresina 64049-550, Piaui, Brazil; tec.heldeney@ufpi.edu.br (H.R.S.); i.dglan@hotmail.com (I.S.L.); luslima_neris@hotmail.com.br (L.M.L.N.); albeertsan@gmail.com (A.S.S.); ariane.am42@gmail.com (A.M.S.S.N.); araujofp15@gmail.com (F.P.A.); durcileneas@gmail.com (D.A.S.); josyosajima@ufpi.edu.br (J.A.O.); 2 Graduate Studies in Agronomy, Mato Grosso of Soulth Federal University, Chapadão do Sul 76560-000, Mato Grosso do Sul, Brazil; rfratke@gmail.com; 3 Research Center on Biodiversity and Biotechnolog, Delta do Parnaíba Federal University, Parnaíba 64202-020, Piaui, Brazil; 4 Veterinary Medicine Academic Unit, Campina Grande Federal University, Patos 58708-110, Paraíba, Brazil; leilson@ufpi.edu.br

**Keywords:** interpenetrating polymer network, controlled release fertilizer, agriculture

## Abstract

Agricultural production is influenced by the water content in the soil and availability of fertilizers. Thus, superabsorbent hydrogels, based on polyacrylamide, natural cashew tree gum (CG) and potassium hydrogen phosphate (PHP), as fertilizer and water releaser were developed. The structure, morphology, thermal stability and chemical composition of samples of polyacrylamide and cashew tree gum hydrogels with the presence of fertilizer (HCGP) and without fertilizer (HCG) were investigated, using X-ray diffractometry (XRD), Fourier Transformed Infrared Spectroscopy (FTIR), Scanning Electron Microscopy (SEM), Thermogravimetric Analysis (TGA/DTG) and Energy Dispersive Spectroscopy (EDS). Swelling/reswelling tests, textural analysis, effect of pH, release of nutrients and kinetics were determined; the ecotoxicity of the hydrogels was investigated by the *Artemia salina* test. The results showed that PHP incorporation in the hydrogel favored the crosslinking of chains. This increased the thermal stability in HCGP but decreased the hardness and adhesion properties. The HCGP demonstrated good swelling capacity (~15,000 times) and an excellent potential for reuse after fifty-five consecutive cycles. The swelling was favored in an alkaline pH due to the ionization of hydrophilic groups. The sustained release of phosphorus in HCGP was described by the Korsmeyer–Peppas model, and Fickian diffusion is the main fertilizer release mechanism. Finally, the hydrogels do not demonstrate toxicity, and HCGP has potential for application in agriculture.

## 1. Introduction

Hydrogels are three-dimensional polymeric networks that have the ability to retain a large amount of the water and maintain their structural integrity [1,2,3,4]. These water-dilated networks are usually formed by polymers, which undergo physical or chemical crosslinking [5]. This high swelling capacity of hydrogels is due to the presence of hydrophilic groups, such as -OH, -COOH, and -SO_3_H, that are connected to the polymeric backbone [6,7,8]. The water absorbed by the material is stored in the empty spaces between the intercrossed polymeric chains that form the hydrogels. When the swelling capacity of hydrogels is very superior (above 100.0%, for example), these materials are named superabsorbent hydrogels (SAHs).

In hydrogels, the three-dimensional structure is formed through covalent bonds (chemical hydrogels) or intermolecular interactions (physical hydrogels) [9]. It is also possible that these two types of interactions occur simultaneously [10]. For chemical hydrogels, the three-dimensional network is formed by polymerization and parallel crosslinking of monomers in the presence of a suitable crosslinking agent [11]. In the case of physical hydrogels, the intercrossing of the chains is formed only by the physical interactions between the macromolecules. In the case of physical hydrogels, the intercrossing of the chains is formed only by the physical interactions between the macromolecules which can occur in a variety of ways, including hydrophobic association, ionic interaction and hydrogen bonding [1,10].

The term hydrogel was first described by Graham in 1864 in his studies of colloidal systems of silicic acid [12]. Over the years, these materials have received notoriety in different fields, including biomedical and pharmaceutical applications [13,14], tissue engineering [15,16], water purification [17], and agriculture [18,19], among others. In agriculture applications, the use of hydrogels contributes to sustainable development and contemplates some of the objective and goal plans set out in the United Nations 2030 agenda. This is because hydrogels, especially SAHs, can store large volumes of water and minimize the waste that occurs with continuous irrigation. The maintenance of humidity in soil also favors a faster germination of the plant, improving the performance of agricultural cultivation [20]. In addition to water reserve systems, SAHs can also be used for the controlled release of fertilizers, which are substances that play an important role in soil fertility [21]. It is estimated that a significant percentage of fertilizers are not completely absorbed by the soil due to the high solubility of these substances in water [22]. For example, about 80.0%–90.0% of phosphorus cannot be absorbed by plants [23], also representing a great waste for agricultural systems. Thus, SAHs combined with fertilizer can improve plant nutrition, reduce water evaporation losses, minimize environmental pollution, and economic losses [24].

SAHs can be of the natural and synthetic type, depending on the polymers used in the synthesis process. Most commercial hydrogels are obtained from synthetic polymers, as they have excellent physical, chemical and mechanical properties [25]. The main polymers used in obtaining synthetic hydrogels are polycaprolactone, poly(vinyl pyrrolidone) (PVP), poly(lactic acid) (PLA), poly(ethylene glycol) (PEG), poly(vinyl alcohol) (PVA) and polyacrylamide (PAM) [26]. PAM is a conventional hydrophilic polymeric substance obtained through the polymerization of acrylamide, which takes place in three stages: initiation (generation of the active center), propagation (growth of the chain with transfer of active centers) and termination (interruption of polymerization due to the disappearance of the active center) [27].

The initiation of polymerization occurs through the use of thermally unstable initiators, which decompose into two active centers (radicals). Immediately, the active radical attacks the double bond of the monomer, transferring the active center and starting the polymerization. The polyacrylamide hydrogel is obtained in two simultaneous steps, which are polymerization and cross-linking. In the case under study, the redox initiator system used is composed of TEMED (tetramethylenediamide) and potassium persulfate (KPS) and the crosslinking agent is *N*′,*N*′-methylenebisacrylamide (MBA) [27].

PAM is a conventional hydrophilic polymeric substance that is used to obtain SAHs, mainly for application as soil conditioner [28,29]. Although PAM exhibits hydrogel formation capacity, chemical treatments, such as alkaline hydrolysis, have been suggested as a way to enhance the swelling of this synthetic polymer [29]. In general, SAH-based synthetic polymers have advantages, such as high swelling capacity and high durability [28]. However, synthetic polymers cannot be degraded in soil and this can represent a serious risk of environmental contamination [8].Thus, natural polymers have stood out as an option to reduce these disadvantages presented by synthetic polymers, and within natural polymers, polysaccharides have been shown to be an excellent option for the formation of SAHs.

Natural polysaccharides exhibit excellent biocompatibility, are biodegradable, are of a class distinct from natural polymers and have a large variety of structural characteristics [26]. Currently, they are being considered in the process of obtaining SAHs due to their economic and ecological viability [18]. In addition, these macromolecules improve the biodegradability, hydrophilicity and biocompatibility of hydrogels [30]. Some studies have simulated the use of different polysaccharides in obtaining hydrogels, such as cellulose [31], alginate [32], gelatin [29], and chitosan [33]; different gums are also related [34]. Natural gums are polysaccharides that can be obtained from exudates and seeds of terrestrial plants, algae and microorganism fermentation. However, plant gums are versatile and have a unique structure and functionality, being used in obtaining materials to ensure an eco-friendly synthesis route [35]. These polysaccharides have a heterogeneous composition and rheological properties that cannot be easily imitated by synthetic polymers [6]. In the presence of water, gums hydrate to form gels whose strength depends on the structure and concentration of the polysaccharide and factors such as pH, temperature and ionic strength [36].

Some studies have explored obtaining SAHs based on tree gums, such as arabic [37], tragacanth [38], ghatti [39], and cashew tree gum (CG) [40]. However, some gums are imported from other continents and this causes an additional cost for the production of technologies. Thus, the use of typical natural polysaccharides in a given region can facilitate the production of new materials, in addition to being an ecologically friendly strategy. The CG is a natural exudate obtained of from the species *Anacardium occidentale L.* [41], which is found in the northeastern region of Brazil [42]. This gum has been used as a substitute for arabic gum, mainly in pharmaceutical applications [43]. Additionally, the extraction and purification of CG is an ecologically correct process [44], representing a sustainable alternative for obtaining new materials for agriculture. The CG consists of a mixture of saccharides instead of a long organic chain formed from chemically bonded units, such as galactose (72–73%), glucose (11.0–14.0%), arabinose (4.6–5.0%), rhamnose (3.2–4.0%) and glucuronic acid (4.7–6.3%) [42]. Industrially, CG has applications in the food and pharmaceutical sectors, mainly as thickening, emulsifying and stabilizing agents [45]. Although some studies have reported the use of CG in obtaining SAHs [27,40,46], these materials were investigated only with respect to the structural, morphological and, obviously, water retention capacity.

In view of these considerations, SAHs play an important role for agricultural production. Therefore, in the present study, a new SAH based on CG and PAM was obtained for the controlled release of water and fertilizer. The swelling performance of SAHs has been investigated under different conditions, and the release of phosphorus as a nutrient was also investigated. These investigations should guide the application of this technology in agriculture.

## 2. Results and Discussion

### 2.1. Structural and Morphological Characterization

**Figure 1:**
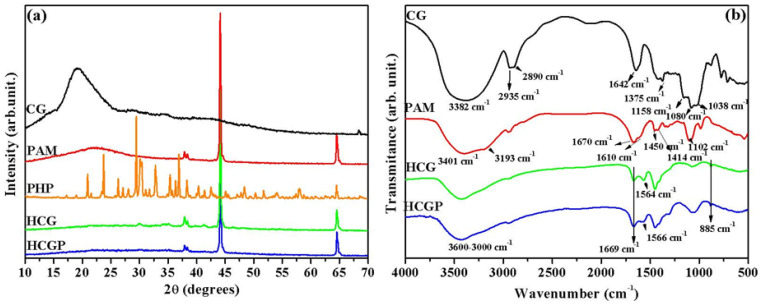
Structural characterization of hydrogel samples and isolated constituents by (**a**) XRD and (**b**) FTIR analysis.

The X-ray diffraction patterns of the CG, PAM, PHP fertilizer, HCG and samples are shown in Figure 1a. According to XRD for CG, the broad diffused center at 2θ = 19.5° indicates a material with a low degree of structural organization, as reported by the literature [47]. The XRD pattern of the PAM showed a discrete, broad diffused center at 2θ = 17.2 that is associated with the amorphous nature of the polymeric matrix [48]. Others’ peaks were observed at 2θ = 44.1 and 64.5°, suggesting the formation of a semicrystalline structure. Similar results were reported by Azzam et al. (2018), who synthesized PAM by the free radical polymerization method [49]. In a PHP diffractogram, a typical crystalline profile was identified, with peaks being observed at different values of 2θ. All these peaks are in good agreement with standard COD 96-200-9611. For HCG and HCGP dry hydrogels, the diffractograms profile demonstrated as similar to the XRD of the PAM. However, the broad diffused associated the amorphous fraction of the PAM and CG polymers were not identified in the hydrogel samples. Peaks associated with PHP were not identified in the HCG and HCGP diffractograms.

The crystallinity index (CrI) and variation of the crystallinity index (∆CrI) were determined for the HCG, HCGP and PAM samples, as seen in Table 1. The HCG and HCGP hydrogels showed an increase in CrI values when compared to the value obtained for PAM. This suggests an increase in the crystallinity of HGC and HGCP samples, which may be associated with the interactions between the CG and PAM chains for the formation of hydrogels [50]. A ∆CrI value of 8.59% and 2.44% was observed for HCG and HCGP, respectively. According to the results, HCG demonstrated greater crystallinity compared to the hydrogel with fertilizer. The reduction in crystallinity may be a consequence of the increase in crosslinking network and a possible distortion in the polymeric network, due to the presence of PHP [50,51].

The functional groups present in the materials were investigated by FTIR analysis, and these results are shown in Figure 1b. The FTIR spectrum of CG revealed the presence of bands at 3382 cm^−1^ that are associated with stretching the O–H bond in hydroxyl groups [52]. The bands at 2935 and 2890 cm^−1^ are referent to C–H stretching (asymmetric and symmetric, respectively), while the bands present at 1642 and 1375 cm^−1^ are associated with the deformation of the O–H bond and flexion of the C–H bond, respectively [47]. Other bands evidenced at 1158, 1080 and 1038 cm^−1^ are due to C–O–C glycosidic bonds and O–H bending from alcohols [27,47]. The typical bands of PAM were observed at 3401 and 3193 cm^−1^, corresponding to axial deformation vibrations in the N–H bond [53,54]. The two bands at 1670 and 1610 cm^−1^ are associated with the C=O stretching vibration in amide, and N–H stretching vibration in amide, respectively [27,48]. Others’ bands, identified at 1450, 1414, and 1102 cm^−1^, were attributed to–CH_2_ scissoring, CN and N–H stretching vibrations [55].

The FTIR spectra of the prepared HCG and HCGP dry samples showed bands characteristic of the functional groups of CG and PAM polymers. The band observed in the range between 3600 and 3000 cm^−1^ may be attributed to the overlap of the N–H and O–H stretching vibration [56,57]; the overlapping of the bands may have occurred due to the presence of physically adsorbed water, since this presence greatly expands the band in this region, while the band at 1669 cm^−1^ corresponds to the C=O stretching present in the PAM structure. The decrease in the intensity of this band was noted for HCG and HCGP; this result is probably associated with the hydrolysis reactions, in which the amide groups (CONH_2_) were converted to carboxylate groups (COONa) [58]. In addition, the band associated with N–H stretching vibration in the amide group was shifted to 1564 and 1566 cm^−1^ in the HCG and HCGP spectra, indicating the occurrence of crosslinking reactions [40]. This band shift may be due to interactions between the polysaccharide and PAM chains. It is well known that the formation of hydrogen bonds reduces the strength constants of chemical bonds, shifting the vibrational frequency to lower wavenumbers [59]. The band displayed at 885 cm^−1^ in the HCG and HCGP spectrum corresponds to out-of-plane C-H bending vibrations, which can be associated with the polysaccharide structure. The bands associated with PHP fertilizer were not identified in the HCGP spectrum, due to the overlap between typical bands of vibrations in the phosphate group and the structure of the polymers. As reported in the literature, the bands associated with the stretching vibration in P=O and P–O are expected at 1109 and 819 cm^−1^ [24]. A summary of the main bands and their assignments is described in Table 2.

**Figure 2:**
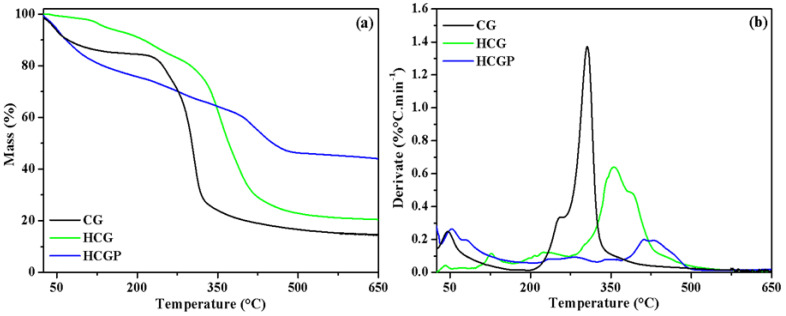
Thermal analysis of CG, HCG and HCGP sample investigated by (**a**) TGA and (**b**) DTG curves.

The thermal behaviors of CG, HCGP and HCGdry samples are shown in Figure 2. Based on the TGA and DTG curves for the CG polysaccharide, the thermal event observed at 56 °C (~13.9% mass loss) is due to water release [47]. Other thermal events that occurred at 254 and 305 °C (69.5% mass loss) are associated with the thermal degradation of the polysaccharide structure [60]. For the HCG sample, four thermal events were observed. The three initial events occurred up to 275 °C (total mass loss at ~6.7%) and are associated with the release of surface water. The last stage of thermal decomposition observed at 354 °C (63.5% mass loss) may be related with thermal degradation of the material.

According to the TGA and DTG curves of the HCGP, the thermal decomposition of this sample occurred in six stages of mass loss. The first two stages below 100 °C (mass loss at 27.4%) may be associated with the release of water or other volatile compounds adsorbed on the material’s surface. The events observed in the range between 200 and 300 °C (with mass loss at 8.7%) and in the region above 350 °C (mass loss at 20.2%) are related to the thermal decomposition of the material. Both for HCG and HCGP samples, the thermal degradation behavior was consistent with the data reported in the literature for polymer based on PAM [61].

Comparing the TGA curves of CG, HCG and HCGP samples, it was observed that mass residue increases in the following order: CG < HCG < HCGP. This result indicates that HCGP had improved thermal stability [29]. The higher thermal stability of the HCGP compared to HCG may be attributed to the formation of crosslinks in the hydrogel matrix [25]. It can be explained that the presence of PHP promoted an increase in the crosslinking reactions during hydrogel formation. In addition, the presence of PHP, which is an inorganic compound that does not degrade, may also have contributed to improvements in the thermal stability of HCGP.

**Figure 3:**
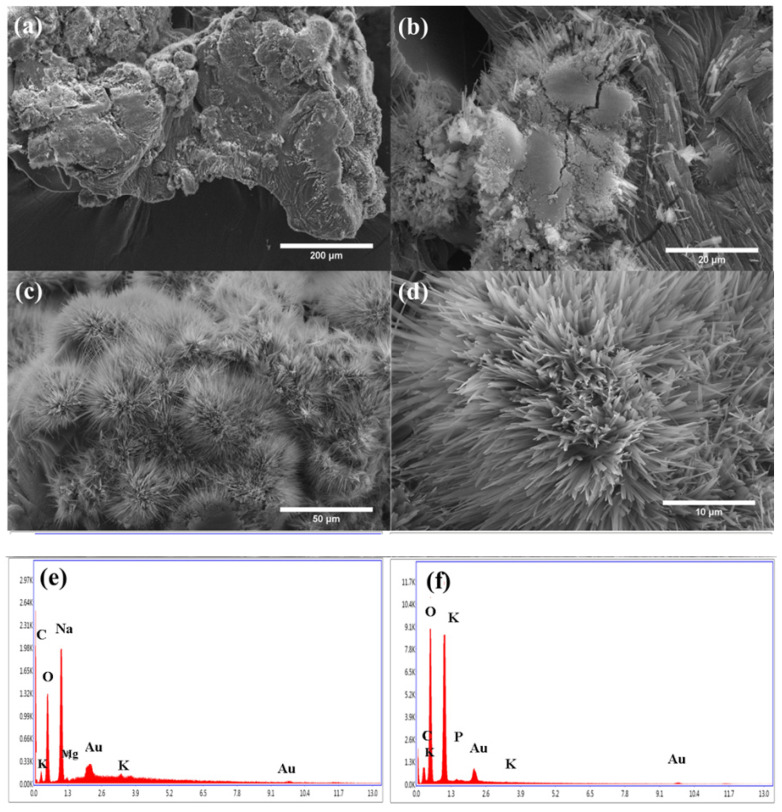
SEM images in different magnifications for samples (**a**,**b**) HCG, (**c**,**d**) HCGP and semi-quantitative analyses by EDS for the (**e**) HCGand (**f**) HCGP samples.

The morphology of the HCG and HCGP was analyzed by the SEM technique in different magnifications, and the results are presented in Figure 3. All the samples were freeze-dried before the SEM morphological analyses. According to these results, HCG (Figure 3a,b) demonstrated surface roughness, being observed in the presence of some agglomerates. Although HCG has demonstrated a more compact surface, this material seems to demonstrate small spaces, probably pores, which should favor the entry of liquids into the hydrogel [8]. Based on the SEM images obtained for the HCGP, it is evident that the addition of PHP completely changed the morphology on the surface material (Figure 3c,d). In this case, a peculiar architecture, formed by fine crystals that grow in parallel, was observed in HCGP. These elongated structures are evenly distributed on the HCGP surface, forming pores that should allow the swelling of the hydrogel [62].

The EDS mapping was performed to analyze the chemical composition of the sample’s surface, and the results are shown in Figure 3e,f. The presence of carbon (C) and oxygen (O) peaks were identified in both the HCG and HCGP spectra and are associated with the organic composition of the materials. In particular, HCG demonstrated a peak related to the sodium element (Na), due to formation of sodium carboxylate groups (COONa) during the hydrolysis process [29]. Peaks associated with potassium (K) and phosphorus (P) atoms were identified in the HCGP spectra and confirm the presence of the PHP fertilizer in this sample.

**Figure 4:**
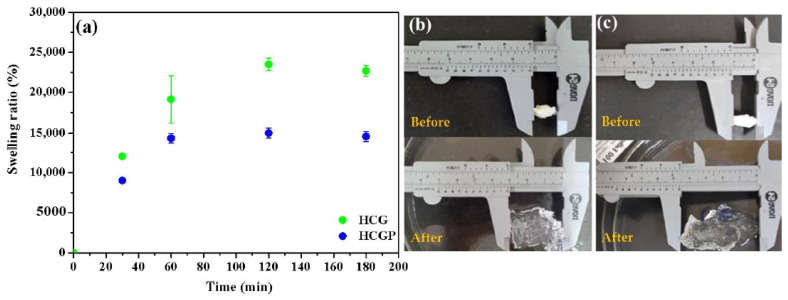
(**a**) Swelling degree as a function of time for the hydrogels samples and physical changes in superabsorbent; (**b**) HCG and (**c**) HCGP before and after the swelling test.

Hydrogels are materials that have a high affinity for water. The water retention capacity of hydrogels is very important to make their application in agriculture feasible. Thus, the swelling of the materials was investigated and the results are shown in Figure 4. In general, the samples showed rapidly swelled in 60 min, and an equilibrium condition occurred in 120 min. According to Figure 4a, it can be seen that the swelling capacity of the HCG hydrogel was greater than that of the HCGP hydrogel. For example, while HCG swelled 24,000 times in relation to the initial mass, HCGP swelled ~15,000 times. It is well reported that the expansion of the hydrogel decreases according to the increase in the crosslink density of the hydrogels [63]. The increase in crosslinking points, causes greater proximity to the three-dimensional networks and, consequently, limits the entry of water into the hydrogel [24,25]. In this way, it is assumed that the addition of fertilizer may have increased the crosslink density in HCGP, resulting in a decreased swelling capacity for this sample. These results corroborated with the thermal stability data presented in Section 2.1. An illustration of the swelling capacity of HCG and HCGP is also shown in Figure 4b,c and prove the efficiency of superabsorbent for both hydrogels. Table 3 summarizes the swelling capacity of other hydrogels based on polysaccharides, as presented in the literature.

**Figure 5:**
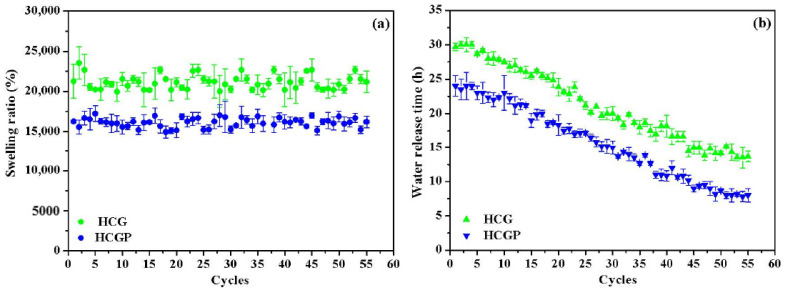
Evaluation of the (**a**) reswelling capacity and (**b**) water release time by HCG and HCGP hydrogels.

In order to examine the potential of HCG and HCGP as soil conditioners, reswelling tests were performed. These results are expressed in relation to the swelling capacity in consecutive cycles and time of water release by hydrogels, as shown in Figure 5. The reswelling capacity of hydrogels after consecutive cycles is an important characteristic to attest the viability of this material in agricultural applications [68]. As observed in Figure 5a, the HCG and HCGP hydrogels demonstrated a constant reswelling capacity during reuse tests. The results suggest that the continuous drying process do not alter the structure of the hydrogels. It is well known that the continuous drying of hydrogels promotes strong interactions of the type of hydrogen bonds between carboxylate and hydroxyl groups present in the polymer chain [69]. Thus, a decrease in network expansion can be expected.

Based on the results of the water release time by hydrogels shown in Figure 5b, a decrease in water elimination time for both hydrogels was demonstrated. For HCG hydrogel, initially, the release of water in the first cycle occurred in 30 h. In the last reuse cycle, the time required to remove water from the structure was 13.5 h. This represents a decrease of 55.0% in the water release time. For the HCGP hydrogel, the water release time in the first cycle was 24 h. At the end of the fifty-fifth cycle, the water release time was 8 h, reducing 67.0% of the time required to dry completely. This behavior can be explained based on the morphology presented by these materials. As demonstrated in the SEM images in Section 2.1, the HCGP showed much finer crystals than the HCG sample. The low thickness of the crystal in HCGP promotes a quick diffusion of water in the structure, facilitating the water release [63]. It is probable that the repeated drying process collapsed the walls of both materials, resulting in a decrease in drying time.

**Figure 6:**
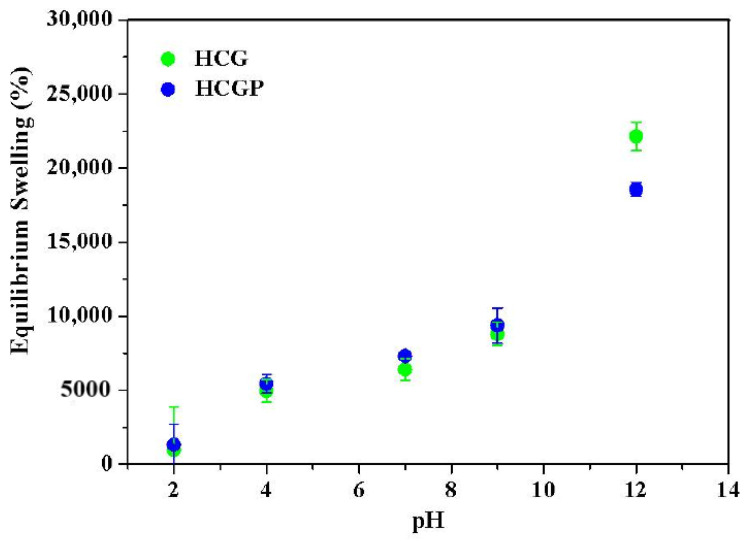
Investigation of the pH-dependent equilibrium swelling ratio of the HCG and HCGP hydrogels.

The swelling property of the hydrogel is strongly dependent on external ambient parameters, such as temperature, pH and ionic concentration [70,71]. In particular, the study on the effect of pH is considered important for application in agriculture, because the soils may have an acidic, neutral or alkaline character. The effect of pH on the swelling behaviors of HCG and HCGP hydrogel was investigated in various pH solutions as displayed in Figure 6. The swelling property of the materials was drastically affected when the pH of the medium was modified. Comparing the swelling capacity at different pH values, it was observed that the swelling ratio in both hydrogels increases with increasing the pH of the solution.

In an acidic pH environment, the swelling capacity was drastically reduced, especially while pH 2 was used for swelling of hydrogels. In this condition, the carboxylate anions (COO^−^) are protonated and this minimize the repulsive anion–anion forces [8,32]. Consequently, the polymeric material becomes more hydrophobic, making swelling more difficult. In addition, hydrogen bonds are favored at an acidic pH and this implies an increase in the degree of physical crosslinking of the chains [25]. Hydrogen bonding type interactions restrict the movement or relaxation of the chains present in the material and form a compact hydrogel network [72]. As discussed, a large number of crosslinking points reduces the physical space for water retention. When the swelling pH of the hydrogels was increased to values above 7.0, a high swelling capacity for the hydrogels was observed. This may have been due to the ionization of hydrophilic groups present in HCG and HCGP in an alkaline pH. The formation of additional physical crosslinking is disadvantaged in situations where the pH is alkaline. In this case, the ionization promotes an electrostatic repulsion between the chains, facilitating the expansion of the hydrogel [25].Consequently, the hydrogels’ swelling capacity was higher in an alkaline medium. The better performance was achieved at pH 12 for all the samples.

**Figure 7:**
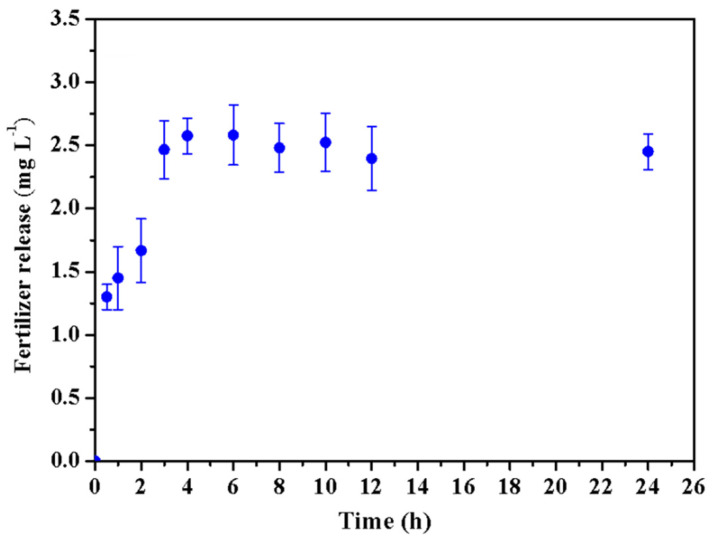
Phosphorus release from the HCGP hydrogel.

Figure 7 displays the release behaviour of phosphorus in HCGP hydrogel. Initially, a rapid increase in the concentration of fertilizer released was observed. This may be associated with the high solubility fertilizer adsorbed on the surface. After four hours, a sustained release was achieved. In the equilibrium condition, the phosphorus concentration found was 2.5 mg L^−1^. BaKi et al. (2018) observed a similar behavior for SHAs based on polyacrylamide grafted with sodium alginate and biochar [73]. The slow release of fertilizer is important to ensure better nutrient absorption and minimize nutrient loss [74]. Thus, HCGP demonstrated a potential for controlled nutrient delivery systems.

**Figure 8:**
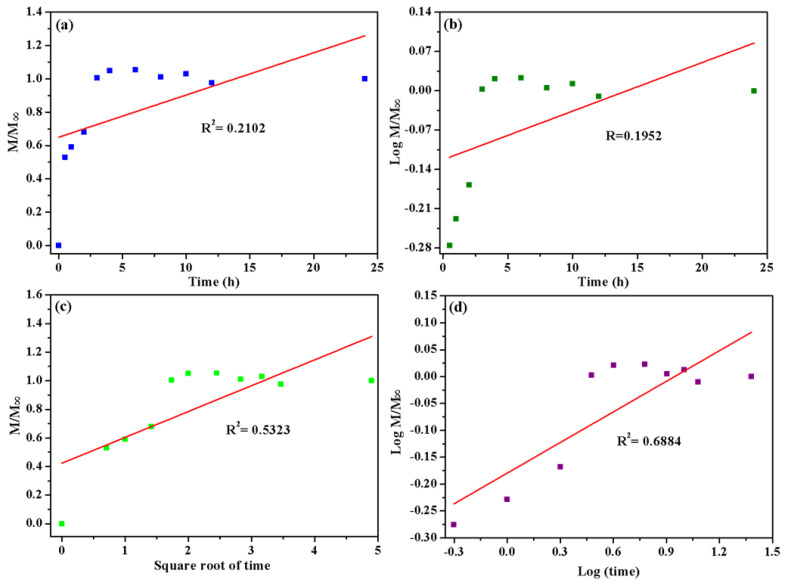
Fertilizer release kinetics according to mathematical models: (**a**) zero order, (**b**) first order, (**c**) Higuchi and (**d**) Korsmeyer–Peppas.

The physical mechanism of fertilizer release by the HCGP hydrogel was determined by comparing the release data with mathematical models of zero order, first order, the Higuchi model and the Korsmeyer–Peppas model. These results are presented in Figure 8. The experimental data did not show a good fit for the zero order and first order models, as indicated by their low regression values (R^2^ = 0.2102 and 0.1952, respectively). It can be observed that the release of fertilizer has higher values of linear regression for the Higuchi and Korsmeyer–Peppas models (R^2^ = 0.5323 and 0.6884, respectively). However, comparing these values with each other, the data have a better fit for the Korsmeyer–Peppas model. To understand the PHP release mechanism, the value of n was determined according to Equation (7), presented in Section 3.6. The value of n was found to be ~0.2. It is well reported that for n < 0.45, the Fickian diffusion mechanism is predominant [75,76], indicating that the diffusion process is the main mechanism involved in transporting PHP molecules through polymeric hydrogel networks [76].

The texture properties are influenced by the composition of hydrogels. In this study, the effect of adding fertilizer on properties such as hardness, deformation, adhesion strength and adhesiveness was investigated, as shown in Table 4. The HGC hydrogel showed higher values for all parameters investigated in the texture analysis. The hardness is crucial to evaluate the durability of a material and, consequently, to ensure better performance during the application [77]. The hardness value of the HCG was 6.2 times superior compared to the HCGP sample. The HGC hydrogel also exhibited superior adhesion strength and adhesiveness values, as seen in Table 4. As noted in the swelling tests, HCG demonstrated a greater ability to retain water than HCGP. Studies are associated with adhesion properties of the increase in crosslink density. More specifically, the existence of hydroxyl groups on the surface that are not crosslinked favors interaction with the substrate and improves the adhesion of the material [78]. In addition, the greater roughness on the HCG surface observed in SEM analysis may also have contributed to improving the sample’s adhesion properties.

### 2.2. Grafting Mechanism

**Figure 9:**
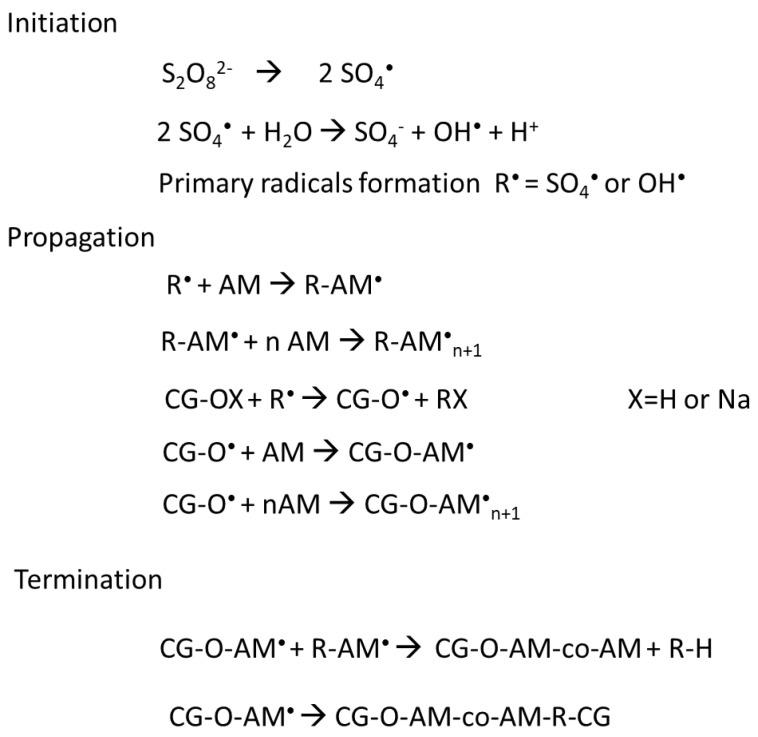
Scheme of copolymerization reaction of the cashew tree range with polyacrylamide, where CG = cashew tree gum; R = Primary radicals (SO_4_^•^ or OH^•^), AM = Acrylamide.

The copolymerization mechanism by grafting the PAM with polysaccharides using redox initiators starts when it is decomposed by heat, generating the sulfate anion radical, which can react with water and generate hydroxyl radicals. Theses radicals are named primary radical (R^●^), which behaves as an active center to initiate the polymerization of PAM [27,79,80,81] as shown in Figure 9. The propagation step follows the reaction of cashew tree gum (CG–OX) with the radicals and monomers. Finally, the termination step occurs when the macroradicals react by themselves, forming the copolymer.

### 2.3. Ecotoxicity

**Figure 10:**
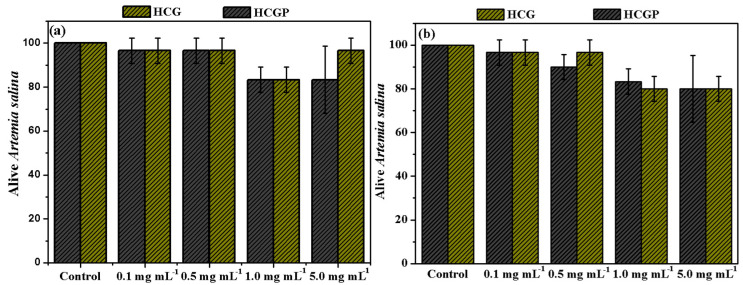
Determination of ecotoxicity in Artemia salina for HCG and HCGP hydrogels after (**a**) 24 and (**b**) 48 h.

To ensure that PAM and CG polysaccharide grafting, as well as the addition of fertilizer, do not promote the toxicity of the final material, ecotoxicity tests in Artermia salina were performed with HCG and HCGP samples. These microcrustaceans occur naturally in aquatic environments, and are sensitive to contaminants even at low concentrations [82]. The results collected during ecotoxicity test are illustrated in Figure 10.

Based on the amount of live nauplii in ecotoxicity tests, the hydrogels demonstrated no toxicity. After 24 h, the amount of the alive nauplii was greater than 80.0% in all concentrations studied. These results are similar to those observed for the control group, where the nauplii were grown in an adequate medium, that is, in saline solution. After 48 h, the amount the alive nauplii remained above 50.0%, confirming the non-toxicity of all the samples. As described by the literature, the CG polysaccharide and PAM do not exhibit significant toxicity [29,83]. Acrylamide is a water-soluble substance and has high toxicity [84]. As evidenced in the results of Figure 9, a survival rate of the nauplii above 80.0% was observed, even after 48 h of contact with extracts of the samples of hydrogels [85,86,87,88,89]. This result also suggests that acrylamide residues do not exist in the samples. Finally, the ecotoxicity results indicate that HCG and HCGP hydrogels do not present risks for use in agriculture.

## 3. Materials and Methods

### 3.1. Materials

The following reagents were used to obtain the hydrogels: Acrylamide, –98.0% (Aldrich, St. Louis, MO, USA); *N*,*N*,*N*′,*N*′–tetramethylenediamine—TEMED, –99.0% (Aldrich), as reaction accelerator; potassium persulfate—K_2_S_2_O_8_–KPS –99.0% (Aldrich) as initiator; crosslinker *N*,*N*′–methylenebisacrylamide—MBA –99.0% (Aldrich); sodium hydroxide—NaOH −97.0% (Aldrich); potassium bicarbonate—KHCO_3_ –99.5% (Aldrich); potassium hydrogen phosphate—K_2_HPO_4_ –99.0% (Exodus);methanol—CH_3_OH –99.8% (Dynamic); ascorbic acid, 99.0% (Aldrich); acetone, 99.5% (Dynamic); ethanol, 99.8% (Dynamic); cashew gum isolated and deionized water were used without previous purification.

### 3.2. Cashew Tree Gum Production

For the isolation and purification of the CG, the procedure previously reported in the literature was used. Initially, the exudates were collected from the tree (Anacardium occidentale L.) located at the Federal University of Piauí-UFPI (SISGEN: ABD61DA). Then, the impurities were removed manually, and lighter and lump-free nodules and impurities were selected. The exudate was dissolved in distilled water (10.0 g in 100.0 mL of water) under mechanical stirring (2000 rpm) for 24 h. The solution obtained was filtered under vacuum and then the pH was adjusted (pH 7.0) with the addition of NaOH solution (0.5 mol L^−1^). The GC was obtained by precipitation of the solution with anhydrous ethanol in a volume ratio of 3:1 (*v*/*v*). The precipitate obtained was centrifuged and washed with acetone. Finally, the gum was kiln dried at 50 °C for 24 h [90]

### 3.3. Hydrogels Synthesis

To obtain hydrogels with fertilizer (HCGP) and without fertilizer (HCG), 2.0 g of CG were dissolved in 30.0 mL of water, under agitation and bubbling with nitrogen gas to reduce the effect of oxygen in the atmosphere on the reaction of polymerization. Then, 0.024 g of K_2_HPO_4_ and 2.10 g of acrylamide monomer were added to the gum solution. After homogenization, 0.016 g of the KPS primer, 0.024 g of the MBA crosslinker and 100.0 mg of KHCO3 were added to the sequence. After 5 min of stirring and bubbling with nitrogen, 100.0 µL of the TEMED accelerator was added to the solution. The system was closed and kept under nitrogen and stirred until the gel point was reached. The obtained gel was washed with a 30.0% (*v*/*v*) aqueous methanol solution to remove residual acrylamide. The material was lyophilized for four days to obtain a dry and constant mass [27].

After drying, the material was subjected to an alkaline hydrolysis reaction for the conversion of its amide groups into carboxylates, increasing the number of hydroxyls, thus making the material more hydrophilic. Thus, 1.0 g of the dry hydrogel was mixed with 40.0 mL of NaOH solution (0.5 mol L^−1^). After finishing this reaction, the materials were washed with deionized water and lyophilized [29].

The fertilizer-free hydrogel (HCG) was prepared similarly to the HCGP hydrogel, but K_2_HPO_4_ was not added during the synthesis process. In addition, PAM was also synthesized in this study.

### 3.4. Characterizations

The dry HCG and HCGP samples were characterized by the X-ray diffraction (XRD) technique (Shimadzu (LABX-XDR 600, Shimadzu, Kyoto, Japan) with Cu-Kα (λ = 1.5406 Å)), using a scan rate of 5° min^−1^. From the XRD data, the relative crystallinity was determined using the crystallinity index (*CrI*) that was calculated by Equation (1):

(1) $$CrI\;\left(\%\right)=\frac{I_{F\;-{\;I}_{S}}}{I_{F}}\times 100$$

where I_F_ and I_F_ correspond to the intensity of the main peak and the secondary peak. The variation of the crystallinity index (∆*CrI*) was determined using Equation (2).

(2) $$\Delta \left(CrI\right)\%=\frac{CrI_{c-CrI_{H\;}}}{CrI_{H}}\times 100$$

where *CrI_c_* and *CrI_H_* are crystallinity index values for copolymer and homopolymers, respectively [50].

The dry hydrogels also were analyzed by Fourier Transformed Infrared Spectroscopy (FTIR). The spectra in KBr pellets were obtained using a Spectrometer (Brucker Optics—Vertex 70, Brucker, Billerica, MA, USA.) in a scanning range from 400 to 4000 cm^−1^ with 64 scans and 4 cm^−1^ resolution. Thermogravimetric analyses (TGA) were performed on an SDT Q600 (V20.9 Build 20, TA Instruments, New Castle, DE, USA.) instrument, using a heating rate of 10 °C min^−1^ (25 to 650 °C), under argon atmosphere with 100 mL min^−1^ in an alumina sample port. The morphological characteristics of the materials were analyzed by Field Emission Scanning Electron Microscopy (FESEM) equipment (QUANTA 250 FEI, FEI Company, Eindhoven, the Netherlands) coupled with elemental analysis by Energy Dispersive Spectroscopy (EDS) (EDAX Apollo X, FEI Company, Eindhoven, The Netherlands). The FESEM images were processed using Image J software.

### 3.5. Swelling Tests

#### 3.5.1. Determination of Swelling/Reswelling Capacity

For swelling tests, 0.20 g of dry HCG and HCGP were immersed in 100.0 mL of distilled water, at an ambient temperature of 25 °C (±2°). After the swelling was balanced, the swollen samples were removed from the medium and the excess water removed with filter paper. Then, the swelling ratio for each sample was determined according to Equation (3) [25]:

(3) $$Swelling\;ratio\;\left(\%\right)=\frac{We-Wo}{Wo}\times 100$$

where *W_o_* and *W_e_* correspond to the hydrogel weights before and after swelling, respectively.

To investigate the reusability of the HCG and HCGP hydrogels, the dry samples were immersed in water (100 mL) at room temperature for swelling equilibrium. The swollen hydrogels were weighed, and the swelling ratio was determined according to Equation (1). Then, the samples were dried at 50.0 °C until they reached constant mass, and a next swelling experiment was performed. This procedure was repeated for up to 55 cycles to determine the reswelling capacity of the samples.

#### 3.5.2. pH Response

To investigate the pH influence on the swelling tests of the samples, the hydrogels were swelled with buffer solution with pH 2, 4, 7, 9 or 12. In each case, the water retention capacity was determined considering Equation (3).

### 3.6. Fertilizer Release Experiment

The controlled release of macronutrient phosphorus in the HCGP sample was performed using a colorimetric assay. For this, 0.20 g of HCGP sample was swelled under conditions similar to those described in the swelling tests. Then, 5.0 mL of the supernatant were removed and mixed with 100.0 mL of the extraction solution in Melich (HCl 0.05 mol L^−1^ and H_2_SO_4_ 0.0125 mol L^−1^), under mechanical stirring for 5 min. Subsequently, a 25 mL aliquot was removed and 10 mL of ammonium molybdate solution was added to it. After this process, 30.0 mg of ascorbic acid was added to the solution and stirred for about 2 min; the system was left to rest for 60 min. Then, aliquots were analyzed by the UV-Vis spectrophotometer at a wavelength of 660 nm, and the release of the fertilizer was quantified [91].

To investigate the kinetics of fertilizer release from the HCGP hydrogel, the following mathematical models were considered: zero-order [22,92], first-order [22], Higuchi model [22], and Korsmeyer–Peppas model [22,93], as shown in Equations (4)–(7):

(4) $$\mathrm{Zero}-\mathrm{order}:\;\left(\frac{M_{t}}{M_{\infty }}\right)=K\;t$$

(5) $$\mathrm{First}-\mathrm{order}:\;\log\left(\frac{M_{t}}{M_{\infty }}\right)=\log\;K+n\;\log\;(t)$$

(6) $$\mathrm{Higuchi}\;\mathrm{model}:\;\left(\frac{M_{t}}{M_{\infty }}\right)=KH_{t}{}^{\frac{1}{2}}$$

(7) $$\mathrm{Korsmeyer}–\mathrm{Peppas}\;\mathrm{model}:\;\left(\frac{M_{t}}{M_{\infty }}\right)=K\;t^{n}$$

where *M_t_/M_∞_* corresponds to the fraction of fertilizer released at a certain time (*t*)*, n* is an indicator of nutrient release mechanism and *K* represents the release kinetic constant.

### 3.7. Texture Analysis

The texture analysis of the hydrogels was performed using a Brookfield CT3 Texture Analyzer. The probe of the type TA-AACC36 with a load of 5 g with a speed of 2 mm s^−1^ under a base of the type TA-BT-KIT was used during the test. The specimens were made using 0.20 g of hydrogels (dimension of 5.0 cm × 4.0 cm) in contact with 50.0 mL of deionized water for a period of 24 h at room temperature.

### 3.8. Toxicity

The toxicity of the HCG and HCGP samples was performed using *Artemia salina*, according to the methodology proposed by the literature. For this, microcrustacean larvae were hatched in 48 h under controlled lighting and oxygenation in a synthetic saline solution (12 ppm) composed of 15.153 g of NaCl, 1.398 g of MgCl. 1.888 g of MgSO_4_, 0.652 g of CaCl_2_, 0.414 g of KCl and 0.116 g of NaHCO_3_ in 1.0 L of distilled water. Then, an amount of 10 live nauplii was collected and placed in contact with the aqueous extracts of HCG and HCGP in concentrations of 0.1, 0.5, 1.0 and 5.0 μg mL^−1^. During the tests, the systems were kept under controlled lighting conditions. After 24 h and 48 h, the microcrustacean survival rate was determined. Synthetic saline was used as a negative control [94].

## 4. Conclusions

SAHs were synthesized from the grafting of CG polysaccharide with PAM. The XRD, FTIR and TGA techniques confirmed the formation of crosslink in the HCG and HCGP samples. The addition of the PHP fertilizer affected the crystallinity, morphology, swelling capacity and properties, such as hardness and adhesiveness, in the HCGP hydrogel. The increase in pH favored swelling due to the ionization of the hydrophilic groups present in the polymeric chain of the material. The sustained release of fertilizer occurred through a diffusion process, as confirmed by fit of the data to the Korsmeyer–Peppas model. In general, the HCG and HCGP materials demonstrated good swelling capacity in consecutive cycles, and the samples exhibited no toxicity. Finally, the results showed that HCGP is promising for use as a water reserve system and the controlled release of nutrients.

## Figures and Tables

**Table 1:** Values of CrI and ∆CrI determined for HCG and HCGP hydrogels.

Sample	CrI	(∆CrI)%
PAM	77.98	--
HGC	84.69	8.59
HGCP	79.89	2.44

**Table 2:** FTIR bands and their assignments for CG, PAM, HCG and HCGP.

Sample	Wave Number (cm^−1^)	Functional Group/Chemical Bond
HCG	3382	Stretching O–H bond in hydroxyl groups
	2935 and 2890	C–H stretching
	1642 and 1375	Deformation O–H bond and flexion C-H bond
	1158, 1080 and 1038	C–O–C glycosidic bonds and O–H bending from alcohols
PAM	3401 and 3193	Axial deformation vibrations in N–H bond
	1670 and 1610	C=O stretching vibration in amide and N–H stretching vibration in amide
	1450, 1414, and 1102	–CH_2_ scissoring, CN and N–H stretching vibrations
HCG and HCGP	3600–3000	Overlap of the N–H and O–H stretching vibration
	1669	C=O stretching
	1564 and 1566	N–H stretching vibration in amide group
	885	Out-of-plane C–H bending vibrations

**Table 3:** Swelling capacity of hydrogels based on other polysaccharides.

Hydrogel Type	Swelling Capacity (g/g)	Reference
Tamarind kernel gum	648	[8]
Guar gum	625	[58]
Agar	14	[62]
Lignin	280	[64]
Galactomannan	115	[65]
Acrylic acid	5066	[66]
Agarose	15	[67]
Starch	921.8	[68]
Cashew tree gum	240	This work
Cashew tree gum with fertilizer	150	This work

**Table 4:** Textural analysis for HCG and HCGP hydrogels.

Sample	Hardness (g)	Adhesion Strength (g)	Adhesiveness (mJ)
HCG	1345.0	45.0	9.4
HCGP	215.0	25.0	1.4

## Data Availability

The data presented in this study are available upon request to the author for correspondence.

## Sample Availability

Samples of the all compounds are available from the authors.
